# Exploring the role of transtibial prosthetic use in deep tissue injury development: a scoping review

**DOI:** 10.1186/s42490-020-0036-6

**Published:** 2020-01-29

**Authors:** Marisa Graser, Sarah Day, Arjan Buis

**Affiliations:** Department of Biomedical Engineering, University of Strathclyde, Graham Hills Building, 40 George Street, Glasgow, G1 1QE Scotland, UK

**Keywords:** Pressure ulcer, Leg prosthesis, Soft tissue injuries, Biomechanical phenomena, Risk factors

## Abstract

**Background:**

The soft tissue of the residual limb in transtibial prosthetic users encounters unique biomechanical challenges. Although not intended to tolerate high loads and deformation, it becomes a weight-bearing structure within the residuum-prosthesis-complex. Consequently, deep soft tissue layers may be damaged, resulting in Deep Tissue Injury (DTI). Whilst considerable effort has gone into DTI research on immobilised individuals, only little is known about the aetiology and population-specific risk factors in amputees. This scoping review maps out and critically appraises existing research on DTI in lower-limb prosthetic users according to (1) the population-specific aetiology, (2) risk factors, and (3) methodologies to investigate both.

**Results:**

A systematic search within the databases Pubmed, Ovid Excerpta Medica, and Scopus identified 16 English-language studies. The results indicate that prosthetic users may be at risk for DTI during various loading scenarios. This is influenced by individual surgical, morphological, and physiological determinants, as well as the choice of prosthetic componentry. However, methodological limitations, high inter-patient variability, and small sample sizes complicate the interpretation of outcome measures. Additionally, fundamental research on cell and tissue reactions to dynamic loading and on prosthesis-induced alterations of the vascular and lymphatic supply is missing.

**Conclusion:**

We therefore recommend increased interdisciplinary research endeavours with a focus on prosthesis-related experimental design to widen our understanding of DTI. The results have the potential to initiate much-needed clinical advances in surgical and prosthetic practice and inform future pressure ulcer classifications and guidelines.

## Background

Pressure injuries frequently affect individuals in hospitals, homes, and care settings. They are defined as “localised damage to the skin and underlying soft tissue”, and are caused by “intense and/or prolonged pressure or pressure in combination with shear” [1]. The severity of this damage can range from superficial, skin bound deteriorations, to full thickness tissue loss. Whilst severely impairing the well-being of affected patients, pressure injuries also pose a tremendous burden on the health care system. In the UK, the estimated cost for pressure ulcer management amounts to £ 507–531 million p.a [2]..

This high number reflects the shortfalls in the prevention, diagnosis, and treatment of pressure injuries, which may be ascribed to ambiguities about their aetiology [3]. An example for ongoing controversies is the recent addition of Deep Tissue Injuries (DTI) to the established pressure ulcer staging system of the National Pressure Ulcer Advisory Panel (NPUAP) [1]. It has long been the prevailing opinion that pressure injuries originate at superficial layers and may evolve into deeper tissues [4]. Accordingly, the NPUAP defined pressure injury stages based on their visual appearance from 1, representing superficial damage, to 4, describing full depth tissue loss [5]. However, an increasing body of literature supports the theory of an opposite route of development [4, 6–9]: Researchers now believe that most pressure-related damage initiates in deep tissues at the bone-muscle interface, from where it may progress towards superficial layers in a bottom-up pathogenesis [10, 11]. Based on its origin, this type of injury is classified as DTI.

The aetiology of DTI is affiliated with a variety of biomechanical, physiological, and biochemical processes on cell and tissue level [7, 12]. In brief, four mechanisms of injury have been postulated to date:

- **Direct deformation** can disrupt the integrity of skeletal muscle cells if the applied load is sufficiently high or prolonged.
- **Ischemia** is caused by a collapse of blood vessels, which obstructs the blood flow and therefore the cells’ oxygen and nutrient supply.
- **Ischemia reperfusion** describes the phase following load removal, where the reoxygenation of the tissue exacerbates ischemic damage, mainly due to oxidative stress.
- **Impaired lymphatic drainage** is induced by obstructed lymphatic vessels, which inhibits the removal of potentially damaging substances from the interstitial space and creates a toxic cell environment.

All these mechanisms contribute to the onset of inflammatory cascades and ultimately cell death. In healthy individuals the cellular damage is usually balanced out by regenerative processes. For certain populations, however, this equilibrium between stress-induced damage and recovery may be disturbed. Accordingly, researchers defined several internal and external determinants of DTI development for a number of risk groups like patients with spinal cord injury (SCI) and wheelchair users [13–19].

Conversely, other populations such as transtibial amputees have gained less attention. During an amputation surgery, foot, ankle joint, and distal tibia and fibula are removed. To restore the capabilities of the amputee, a prosthesis is fitted to the residuum, which transfers forces between the ground and the body’s support structures during ambulation. The soft tissues that cover the stump become part of this load bearing system, even though they are not physiologically adapted to withstand high external forces. Consequently occurring skin problems, pain, and ulceration can lead to rejection of the prosthesis [20–23].

Whilst the prevalence and incidence of superficial wounds is well documented [20, 22], only few case studies have recorded DTI occurrence [24, 25]. A lack of knowledge, ambiguities in classification, and the absence of established reporting procedures [26] may play into this. To provide a basis for much-needed advances in the prevention, diagnosis, and treatment of DTI, a better understanding of the underlying aetiology and risk factors is necessary.

We therefore aim to map out and critically discuss research on DTI in transtibial amputees as a result of prosthetic use. Our focus is on (1) the population specific aetiology, (2) risk factors, and (3) methodologies to quantify and measure both. Because randomised controlled trials are sparse in rehabilitation science [27], we chose to conduct a scoping review. The systematic approach allowed for the assessment of the nature and extent of various types of research and to identify gaps in the literature. We hope that connecting the multifaceted challenges of transtibial prosthetic users with the aetiology of DTI will provide the basis for future research in this area. Additionally, our results could inform prospective policies, guidelines, risk assessment tools, and international classifications.

## Results

### Search results and grouping

**Fig. 1:**
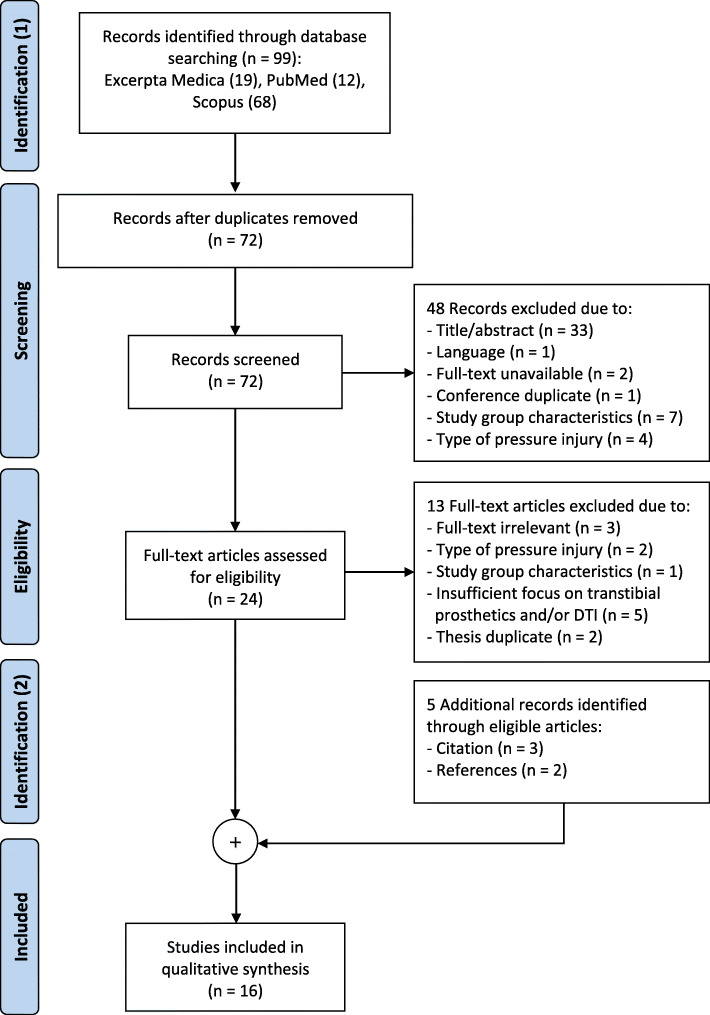
PRISMA flow chart for literature search process and results

The automated database search on the 14th of June 2019 resulted in 19 identified sources from Ovid Excerpta Medica, 12 from PubMed, and 68 from Scopus. Removal of duplicates left 72 articles for the screening process. After applying inclusion and exclusion criteria, 24 articles remained for full-text assessment, of which 11 met the eligibility criteria. An additional 5 articles were identified through references and forward-citation of these sources, amounting to 16 studies in total (Fig. 1).

**Fig. 2:**
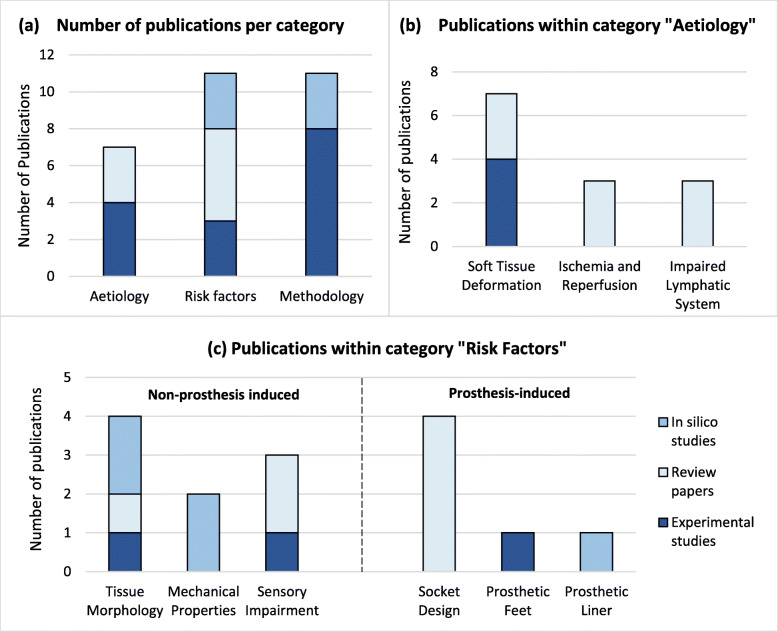
Number of publications per category. **a** Publications per main category (aetiology, methodology, risk factors). **b** Publications within category “Aetiology”. **c** Publications within category “Risk Factors”, subdivided into “Non-prosthesis induced” and “Prosthesis-induced”. The types of papers analysed include reviews (medium blue), in silico studies (light blue), and experimental studies (dark blue). Some papers matched multiple groups, thus the total number of publications in this figure exceeds the number of studies analysed

The 16 identified articles covered a variety of study types. Eleven of them presented primary research, of which seven examined human subjects experimentally [28–34] and one tested ex vivo animal tissue [35]. Three papers were in silico studies [36–38]. The other five sources were secondary articles in the form of literature [9, 26, 39, 40] or systematic reviews [41]. They were all thematically grouped by research focus into the following categories (Fig. 2):

1. DTI aetiology in transtibial prosthetic users
2. Risk factors for transtibial prosthetic users
3. Methodological approaches to measure aetiological and risk factors in transtibial prosthetic users

### Aetiology

Of the 16 articles, 44% (7) discussed the aetiology of DTI, focussing mainly on residuum biomechanics. Between 2007 and 2011, Portnoy and colleagues conducted four experimental studies [29, 31, 33, 34] on a total of 26, unilateral, transtibial amputees. The participants were typically 44 years old, male (85%), weighted 71.72 kg, and presented with trauma as cause of amputation (59%) (Table 1). The researchers aimed to quantify the internal loading states of deep muscle layers during scenarios encountered by prosthetic users in their daily life: Dynamic walking on various surfaces [29, 31], static weight bearing during standing [34], and prolonged sitting [33].

**Table 1:** Experimental studies on the deformation as aetiological factor for DTI in transtibial prosthetic users

Author and year	Objective	Loading scenario	Study Population	Quantitative Results	Qualitative results
Portnoy et al. 2007 [29]	Test of feasibility of real-time FE^a^ monitor to estimate internal tissue load	Dynamic load: Treadmill walking	- 5 unilateral TTA^b^s - 1 female, 4 male - Mean age 47.2 yrs. - Mean weight 68 kg	Max. principal compression stresses: - Fibular axis: 16.38 kPa (3.5 - 31 kPa) - Gastrocnemius axis: 8.19 kPa (1.75 - 13 kPa)	- Maximum compressive stress between midstance and push-off - High inter-patient variability in stress magnitude
Portnoy et al. 2008 [34]	Estimation of internal tissue load during static load bearing	Static load: Weight bearing	- 1 unilateral TTA - Female - Age 29 - Weight 50 kg - Traumatic cause	- Compressive stress 240 kPa - Tensile stress 263 kPa - Shear stress 23 kPa - Compressive strain 85% - Tensile strain 129% - Shear strain 106% - SED^c^ 104 kJ/m^3^ - Von Mises stress 215 kPa	- Compression slightly above physiological levels in comparison to gluteal tolerance - High tensile and shear strains which may be risk for tissue viability - Stress and strain concentrations in flap under tibial end
Portnoy et al. 2010 [31]	Determination of subject-specific dynamic stresses in soft tissues	Dynamic load: Walking on complex terrain	- 18 unilateral TTAs - 1 female, 17 male - Mean age 43 yrs. - Mean weight 79 kg - 11 traumatic, 7 vascular	Average peak von Mises stress: - Plane: 100 kPa - Grass: 80 kPa - Upstairs: 95.1 kPa - Up slope: 83 kPa - Downstairs: 115.4 kPa - Down slope: 141.9 kPa	- High inter-terrain and inter-subject variability - Higher stresses in vascular compared to traumatic patients - No immediate risk of DTI for 10 out of 18 subjects - Elevated peak stress when descending stairs or slope compared to plane gait
Portnoy et al. 2011 [33]	Evaluation of risk of DTI development for during sitting with a donned prosthesis	Static loading: Sitting with 30° and 90° knee flexion	- 1 unilateral TTA - Male - Age 55 - Weight 73 kg Traumatic cause	At tibial end (90° flexion): - Principal stress: Compression 102.9 kPa Tension 66.6 kPa - Shear stress 67.2 kPa - Von Mises stress 129 kPa Volume of damaged area (after 75 min): - 30°: 13.5mm^3^ - 90°: 600mm^3^	- Residuum at risk of DTI during sitting with 90° flexion - Risk of DTI posture dependent - Injury rate higher with increased knee flexion - Damage volume may be dependent on muscle thickness

^a^Finite element; ^b^Transtibial amputee; ^c^Strain Energy Density

Across all these scenarios, the researchers located peak loads at the distal end of the bones [29, 31, 33, 34]. During normal gait, the peak was reached between midstance and toe off [29], and its magnitude appeared to be terrain-dependent [31]. When sitting with a donned prosthesis, the load increased simultaneously to the degree of knee flexion [33]. However, high inter-patient variability made the interpretation of absolute values difficult [29]. Portnoy et al. therefore compared individual stress and strain values to loading-thresholds for deformation-induced cell death to assess the risk for DTI development [18, 42, 43]. They hereby surmised hazardous loading conditions during weight bearing [34], walking over complex terrain [31], and sitting with the knee in 90 degree flexion [42]. Overall, DTI appeared to be a threat for several transtibial prosthetic users in everyday situations.

Cellular deformation is only one of several aetiological factors of DTI. Mak et al. [9, 40] and Bader et al. [26] suspected a link between prosthetic use and disruptions of the lymphatic and microvascular system (Table 2). When externally applied pressure and shear deforms the deep tissues of a residuum [29–31, 33], the embedded lymphatic and vascular structures will be distorted as well. However, the extent of this deformation and its consequences have not been investigated on transtibial amputees.

**Table 2:** Literature reviews on the aetiology of DTI in transtibial prosthetic users

Autor and year	Objective	Aetiological factor	Qualitative results
Mak et al. 2001 [40]	Overview over current knowledge about biomechanics in TTA^a^ s	Deformation, impaired lymphatic system, ischemia	- Skeletal movement and friction lead to soft tissue deformation during gait - Stress and deformation affect cellular and tissue function mechanically and via impairment of ancillary systems - Magnitude, direction, distribution, duration, loading rate are crucial - Possible accumulative effect of repetitive stress
Mak et al. 2010 [9]	Overview over tissue response to loading	Deformation, impaired lymphatic system, ischemia, ischemia-reperfusion	- Immediate muscle damage by direct mechanical insult or over longer period by ischemia - Reperfusion exacerbates damage by oxidative stress and inflammation - Damage may accumulate if insufficient recovery time
Bader et al. 2019 [26]	Analysis of bioengineering tools for device related soft tissue damage	Deformation, lymphatic drainage, ischemia, ischemia reperfusion injury	- Direct deformation, impaired lymphatic drainage, disrupted microvasculature and ischemia reperfusion as aetiological factors - High inter-patient variability

^a^ Transtibial amputee

### Risk factors

A total of 68.75% (11) of the identified literature examined risk factors of DTI in transtibial prosthetic users. The population investigated in three experimental and two in silico studies amounts to 33, typically male (90.90%), transtibial amputees with traumatic amputation (84.84%), a mean age of 49 years, and an average weight of 81.1 kg. These studies, together with an additional in silico study and five review papers, were divided by non-prosthesis induced (63.63%, 7) and prosthesis-induced (54.54%, 6) risk factors.

#### Non-prosthesis induced risk factors

Non-prosthesis induced risk factors originate from intrinsic characteristics of the transtibial residuum. According to the literature body, those factors are tissue morphology, the mechanical properties of the soft tissue, and sensory impairments (Table 3).

**Table 3:** Studies on non-prosthesis induced risk factors for DTI

Author and year	Type of Study	Objective	Non-prosthesis induced risk factors	Study Population	Qualitative Results
Henrot et al. 2000 [39]	Literature review	Overview over surgical features of amputation in relation to postoperative stump pain	Tissue Morphology	NA	- Postoperative complications: Heterotopic ossification, aggressive bone edges, pistoning, ulceration - Extrinsic pain: caused by improper fit or alignment - Intrinsic pain: nervous or anatomical cause
Portnoy et al. 2007 [37]	In silico study	Determination of internal stress in muscle flap of TTA^a^; influence of bone sharpness, tissue thickness, mechanical properties	Tissue Morphology,Mechanical Properties	- Use of data from Portnoy et al. 2007 [29]	- Increase in pressure with reduced flap thickness - Increase in pressure with increased muscle stiffness - No clear relationship between bone radius and pressure
Portnoy et al. 2009 [30]	Experimental study	Determination of inter-subject variability in internal tissue loads in TTA with different geometrical features during static loading	Tissue Morphology	- 5 unilateral TTAs - Traumatic cause - 1 female, 4 male - Mean age 48.6 yrs. - Mean weight 67.8 kg	- High inter-subject variability - Risk of DTI for patients with little fat padding - More even pressure distribution with flat compared to sharp tibial ends - Lower strains with longer residua
Portnoy et al. 2009 [36]	In silico study	Identify influence of risk factors on internal loadingonditions in TTA	Tissue Morphology,Mechanical Properties	- 1 unilateral TTA - Traumatic cause - Male - Age 44 yrs. - Weight 82 kg	- Thicker muscle flap and increased bone radius reduce DTI risk - Osteophyte, sharp bevelment and muscle stiffening increase risk - Surgical scars change overall stress distribution
Kosasih et al. 1998 [28]	Experimental study	Identify sensory changes in well healed TTAs	Sensory impairment	- 9 unilateral TTAs - Traumatic cause - All male - Mean age 55.4 yrs. - Weight not specified	- No deep pressure sensory impairment in well healed TTA stumps
Mak et al. 2001 [40]	Literature review	Overview over current knowledge on biomechanics in TTAs	Sensory impairment	NA	- Neuropathy leading to usually preventable soft tissue damage
Bader et al. 2019 [26]	Literature review	Analysis of bioengineering tools for device related soft tissue damage	Sensory impairment	NA	- Risk factors: neuromuscular impairments, diabetes

^a^ Transtibial amputee

Tissue morphology was the most frequently investigated determinant. Henrot et al. [39] and Portnoy et al. [30, 36, 37] focussed on surgery-related and pathological changes in tissue morphology. They identified the following risk factors: Thin muscle flaps [36, 37], minimal adipose tissue padding [30], sharp bone edges [30, 36, 39], and osteophytes [36, 39]. Results on the influence of the bone radius were ambiguous. A numerical in silico study indicated that the soft tissue load is reduced with wider bone radii [36], whilst an analytical study found no relationship between the two [37].

The effect of sensory impairment was discussed by three papers. According to reviews [26, 40] the loss of sensation, as it is commonly seen during diabetes, causes a dysfunction of the natural protective pain mechanism to redistribute excessive loads. Kosasih et al. [28] suspected that sensory changes could also result from the amputation process. However, contrary to their expectations, deep pressure sensation of their test subjects seemed to be intact.

The mechanical properties of soft tissue were examined in two in silico studies. Both indicate a higher susceptibility of stiff muscle to loading damage compared to a flaccid one [36, 37]. This stiffening might be the result of muscle contraction during normal gait [36, 37] or pathological changes like spasticity [36]. Portnoy et al. [36] also suggest that surgical scars change the stress distribution across the residuum. The lower elasticity of fibrous scar tissue may put the surrounding muscle at risk of DTI development.

#### Prosthesis induced risk factors

The prosthesis itself also influences the development of DTI in transtibial amputees. Relevant factors are socket design and prosthetic components (Table 4). The most abundantly discussed prosthetic element was the socket (4). It distributes pressure and shear forces across the limb during weight-bearing [9, 26, 40]. Based on two fundamentally different socket design philosophies, these loads may either be concentrated on proclaimed tolerant areas (Patellar Tendon Bearing (PTB)), or spread as evenly as possible over the entire residuum (Total Surface Bearing (TSB)) [9, 40]. Whilst differences in interface loads between both design approaches have been found [40, 41], the internal loading conditions have yet to be quantified [26, 40, 41].

**Table 4:** Studies on prosthesis-induced risk factors for DTI

Author and year	Type of study	Objective	Prosthesis-induced risk factor	Study Population	Qualitative results
Mak et al. 2001 [40]	Literature review	Overview over current knowledge about biomechanics in TTA^a^ s	Socket design	NA	- Influence of socket shape on pressure distribution - Need to quantify residuum-socket interactions - Need to address controversies in socket design
Mak et al. 2010 [9]	Literature review	Overview over experimental and computational studies on tissue deformation and pressure ulcers	Socket design	NA	- Influence of socket interface on transmission of pressure and shear to residual limb - Pressure profile affected by variations in socket and fitting techniques - Even load distribution as way to reduce DTI risk
Dickinson et al. 2017 [41]	Systematic review	Critical appraisal of state-of-the-art in FE^b^ analysis in TTAs	Socket design	NA	- Influence of socket design and material properties on loading conditions - Clinical translation of FE models necessary
Bader et al. 2019 [26]	Literature review	Review of Medical Device Related Pressure Ulcers (MDRPUs) and technologies for their detection	Socket design	NA	- Deviations between stiffness of device material and skin/subdermal tissue as major issue - Inadequate guidance for use of devices - Individualisation as key concept
Portnoy et al. 2012 [32]	Experimental Study	Assessment of differences between hydraulic and ESR feet on internal loading conditions when walking over complex terrain	Prosthetic feet	- 9 unilateral TTAs - Traumatic cause - All male - Mean age 42.7 yrs. - Mean weight 78.2 kg	- Significant decrease in internal stress and loading rates with hydraulic vs ESR feet when walking on paved floor or ascending stairs - Tendency towards internal stress reduction when using split-toed ESR compared to single-toe version
Lenz 2017 [38]	In Silico Study (PhD thesis)	Investigation of pressure ulcer formation due to deformation, based on gel liner displacement	Prosthetic liner	NA	- Increased interface shear with addition of socks over liner - Cushioning effect of gel liner - Increase in shear stress at bone-muscle interface with no-slip condition - Increase in shear and von Mises but decrease in compression at bone-muscle interface with decreasing liner thickness - Decreased muscle compressive stress with increased liner stiffness

^a^ Transtibial amputee; ^b^ Finite Element

Two papers on prosthesis induced risk factors addressed components other than the prosthetic socket. Across two experimental studies, a total of 18 traumatic, mainly male (94%) and unilateral (94%) amputees with an average age of 49.8 years and weight of 85 kg participated. Researchers thereby investigated the influence of prosthetic foot design [32] as well as gel liners and socks [38] on the internal loading states. Hydraulic and split-toed feet seemed to improve loading conditions in comparison to energy storage and return (ESR) and single-toe feet. Furthermore, the use of gel liners, especially stiff and thin ones, appeared to provide a positive cushioning effect. In contrast, the common practice of adding socks over the liner to accommodate volume fluctuations of the stump was predicted to increase interface shear, which may adversely affect deeper tissues.

### Methodology

All experimental and in silico studies (11) were included in an overview of methodologies in DTI research on transtibial prosthetic users. The studies were grouped by methodological approaches, namely finite element (FE) analysis, analytical modelling, acoustic signalling, and sensory analysis.

#### Finite element analysis

FE analysis is based on computational models of the residuum with defined geometries, material properties, and boundary conditions. These models are numerically analysed to create visual stress and strain maps. With 54.54% (6), FE analysis constitutes the most common methodology in primary research (Table 5). The studies can be divided into three groups: individualised biomechanical models for aetiological research [30, 33, 34], in silico studies for parametric analysis of risk factors [36, 38], and real-time FE for clinical applications [29].

**Table 5:** Studies on DTI in transtibial prosthetic users using FE analysis

Author and year	Type of study	Methodology	Input data	Assumptions	Outcome measures
Portnoy et al. 2008 [34]	Experimental study (aetiological)	- 3D FE^a^ model - Donning and static load bearing of one TTA^b^ - Analysis of internal loading state	- Interface pressure (pressure sensor) - Tissue morphology and vertical displacement (MRI^c^) - Shear modulus, friction between skin and socket (literature)	- Muscle: isotropic, homogenous, viscoelastic - Skin: isotropic, homogeneous, hyperelastic - No differentiation btw. Muscle and fat - No friction between soft tissue layers	- SED^d^, principal compressive and tensile stress and strain, max. Shear stress and strain, von Mises stress
Portnoy et al. 2009 [30]	Experimental study (aetiological)	- 3D FE model [34] - Static load bearing of five TTAs - Analysis of internal loading state and interpatient variability - Evaluation of DTI risk	- See Portnoy et al. 2008	- Soft tissue: isotropic, homogeneous, hyperelastic - Differentiation btw. Muscle and fat - Addition of 2 mm skin layer - No friction between soft tissue layers	- Volume of muscle skin with compressive, tensile, shear strains above threshold value [43]
Portnoy et al. 2011 [33]	Experimental Study (aetiological)	- 3D FE model [34] - Sitting with 30° and 90° knee flexion in one TTA - Assessment of internal loading state and estimation of damage area over time	- See Portnoy et al. 2008	- Soft tissue: isotropic, homogeneous, hyperelastic - Differentiation btw. Muscle and fat tissue - Addition of 1 mm skin layer - No friction between soft tissue layers	- Principal tensile and compressive stress, max. Shear stress, von Mises stress - Time-dependent volume of damaged muscle [43] - Rate of damage progression [44]
Portnoy et al. 2009 [36]	In silico study (risk factors)	- 3D FE model [34] of one TTA - Changes in morphological and mechanical parameters	- See Portnoy et al. 2008	- See Portnoy et al. 2009 [30]	- SED, principal compressive and tensile stress and strain, max. Shear stress and strain, von Mises stress, - Volumes of areas with concentrated elevated stress
Lenz 2017 [38]	In silico study (PhD thesis, risk factors)	- Analysis of internal loading state with simplified cuboid FE model - Simulation of different liners and socks - Differentiation between slip and no-slip condition	- Liner displacement and mechanical properties (motion capturing) - Normal and shear interface forces (two-axis load cell) - Shear modulus, friction between skin and liner, soft tissue and liner thickness (literature)	- Muscle: isotropic, homogeneous, hyperelastic - Differentiation btw muscle, skin, gel liner - No friction between soft tissue layers - Friction between skin and gel liner (slip vs. no-slip)	- Principal compressive stress, max. Shear stress, von Mises stress
Portnoy et al. 2007 [29]	Experimental Study (clinical)	- 2D FE model for real time stress analysis - Application on 5 TTA^a^ s during treadmill walking	- Interface pressure (pressure sensor) - Elastic modulus (Indentation test) - Tissue morphology - (X-Ray)	- Soft tissue: isotropic, homogenous, linear elastic - No differentiation btw. Muscle, fat, and skin	- Principal compressive stress and strain, shear stress, von Mises stress

^a^ Finite Element; ^b^ Transtibial amputee; ^c^ Magnetic Resonance Imaging; ^d^ Strain Energy Density

When investigating residuum biomechanics for aetiological research, individualisation and high accuracy are key [41]. Accordingly, researchers obtained subject-specific morphological and loading data from MRIs and interface pressure sensors. Certain aspects of mechanical properties of soft tissue were also integrated into the analyses, for example viscoelasticity [34] or hyperelasticity [30, 33]. Others, like heterogeneity and anisotropy were disregarded. The resulting computed stress and strain levels were compared to threshold values for deformation-induced cell death to determine the subject-specific DTI risk [30, 33]. For further refinement, damage progression was predicted by iterative post-processing [33], which incorporated muscle stiffening following the initial damage [44].

For risk factor research, in silico modelling is an important tool. By changing single input parameters of an FE model, their effect on internal loading conditions can be tested. Portnoy et al. [36] produced an FE model, following the process described above, before altering bone geometry, mechanical properties of the muscle, and tissue homogeneity. Lenz [38] simulated the use of different liners and liner-socket interface conditions in a slightly different way. Instead of representing a full residuum, she reduced her FE model to a cuboid shape with soft tissue thicknesses taken from literature. The loading conditions were recorded with a two-axis load cell, rather than pressure sensors, and with motion capture data of liner displacements during gait.

To translate these complex models into clinical use, real-time availability of information is desirable. Thus, Portnoy et al. [29] proposed an X-Ray based FE model of the residual limb, with interface pressure measurements and patient-specific elastic moduli feeding into it. Rendering bones as rectangles and assuming linear elasticity, isotropy, and homogeneity was accepted as trade-off between accuracy and computing time.

Overall, FE analysis is a common tool in biomechanical research of DTI development. It provides full field information on the internal loading state of soft tissue and allows to assess the influence of single risk factors. The model complexity, input, and output data vary widely across the different studies.

#### Analytical methods

For clinical applications, two-dimensional analytical solutions, as utilised in three primary studies [29, 31, 32], are an alternative to FE analysis (Table 6). The models evaluate the internal loading state of the residuum mathematically, based on simplified tissue geometries and mechanical behaviour. The first approach developed by Portnoy et al. [37] calculated the contact pressure between bone and soft tissue based on Hertz contact theory. Despite being validated in silico, this model was to the authors’ knowledge never translated to experimental research.

**Table 6:** Studies on DTI in transtibial prosthetic users using analytical modelling

Author and year	Type of study	Methodology	Input data	Assumptions	Outcome measures
Portnoy et al. 2007 [37]	In Silico Study	- Application of Hertz contact theory for calculation of contact pressure between bone and soft tissue - Evaluation of sensitivity of pressure calculations to tibial radius, muscle thickness and mechanical properties	- Tissue morphology (X-Ray [29]) - Poisson’s ratio (literature)	- Soft tissue: isotropic, homogeneous, linear elastic - No friction between bone and soft tissue - Tibia simplified as flat-ended cylinder - Only vertical bone displacement	Contact pressure between tibia and soft tissue
Portnoy et al. 2010 [31]	Experimental Study	- Development of portable monitor based on an axi-symmetric indentation problem - Use of monitor on 18 TTA^a^ s to record internal loads during walking on complex terrain - Comparison of internal loads between patient groups and surfaces	- Interface pressure (pressure sensor) - Tissue morphology (X-Rays) - Shear modulus, friction between skin and socket (literature)	- Soft tissue: isotropic, homogeneous, linear elastic - No differentiation between muscle, fat, and skin - Tibia simplified as flat-ended cylinder	Average von Mises stress, loading rate, stress-time integral
Portnoy et al. 2012 [32]	Experimental Study	- Use of portable pressure monitor [31] on 10 TTAs - Assessment of internal stress during walking on complex terrain - Comparison of outcomes for ESR^b^ foot and hydraulic foot	- See Portnoy et al. 2010	- See Portnoy et al. 2010	Average von Mises stress, RMS^c^ of von Mises, loading rate, cadence

^a^ Transtibial amputee; ^b^ Energy Storage and Return; ^c^ Root mean square

Instead, Portnoy et al. utilised a similar model [45] in their experimental studies [31, 32]. The bone was assumed as an axisymmetric, flat indenter, which compresses a linear elastic soft tissue layer. Individual interface pressure measurements and X-Rays in combination with literature-based values for tissue material properties and boundary conditions provided data on the essential variables. This allowed for a real-time estimation of internal loading states.

#### Acoustic signalling

Following the idea of direct deformation as major aetiological factor, Buis et al. [35] attempted to find ways other than imaging to measure tissue distortion (Table 7). The idea was that deformation would create acoustic signals. In an ex vivo experimental study, they subjected porcine and galline tissue immersed in a saline bath to tensile stress whilst recording acoustic emissions with a hydrophone. However, they were unable to detect sufficient acoustic signals to support their hypothesis.

**Table 7:** Studies on DTI in transtibial prosthetic users using acoustic emission and sensory tests

Author and year	Type of study	Parameter of interest	Methodology	Input	Outcome measures
Buis et al. 2018 [35]	Ex vivo animal study	Acoustic signalling	- Tensile test setup - Immersion of bovine and galline specimen in saline solution - Recording of acoustic signals during tensile test of specimen with hydrophone	Tensile load and displacement (Instron)	Acoustic emission: amplitude and frequency (No correlation found btw. Acoustic emission and deformation)
Kosasih et al. 1998 [28]	Experimental Study	Sensory analysis	- Qualitative sensory assessment of the residuum in 16 TTA^a^ s - Test sides: pressure tolerant and pressure sensitive areas of residuum - Contralateral limb as control	Physical administration of cotton swab wisp, firm pressure, tuning fork vibration, safety pin prick by physician	Qualitative feedback about sensory response either at detection of stimulus or with description (i.e. sharp or dull pain)

^a^ Transtibial amputee

#### Sensory analysis

The only primary study that was not biomechanics related was an experimental study on sensory changes in transtibial prosthetic users [28] (Table 7). A physician applied light touch, deep pressure, vibration, and pin pricks to the residuum as well as the sound limb of participants. The resulting sensory perception for each stimulus was evaluated via qualitative feedback.

## Discussion

In this scope review we identified research and literature on DTI as a result of transtibial prosthetic use. We addressed three key areas: Firstly, the aetiology of DTI in prosthetic users is only partially understood. Whilst direct deformation seems to be an important factor, the contribution of ischemia, ischemia reperfusion, and an impaired lymphatic drainage has yet to be evaluated. Secondly, we identified several risk factors. Intrinsic determinants of DTI damage are tissue morphology and its mechanical properties, as well as sensory impairment of the residuum. Extrinsic determinants are socket design and prosthetic components. Finally, we found that methodological approaches in both aetiological and risk factor research focussed mainly on biomechanics. The most common research design combined static or dynamic loading with medical imaging and computational analysis. However, the diversity of input data, modelling assumptions, and outcome measures, together with the variability in geometrical patient characteristics and prosthetic components make a quantitative comparison of results and their clinical translation difficult.

### Damage pathways

Within the last two decades, major research efforts led to advancements in our understanding of DTI. The pathways of tissue damage from external loading were investigated on different organisational levels, from cell cultures over engineered muscle constructs to in vivo animal studies [7, 46]. The results indicate that the relationship between the degree of deformation and time is essential: Whilst high strain can cause immediate direct deformation damage, moderate strain may injure the tissue by occluding the vascular and lymphatic system over a longer period.

#### Loading biomechanics and direct deformation

The biomechanical profile at the stump-socket interface of lower-limb amputees differs significantly from the frequently studied immobile individuals [15, 16, 18, 47–51]. Prolonged loading is only seen in situations of static weight bearing like standing or sitting with a donned prosthesis. In contrast, with over 8000 steps taken daily by transtibial amputees [52], the residuum often experiences dynamic, cyclic loading. During gait, complex internal stresses and strains develop: weight bearing causes pressure at the socket-limb interface [53]; the vertical pistoning movement between the residuum and the socket invokes shear forces [54]; and gait action and bone movement lead to the development of additional forces and moments [55, 56]. The combination of these forces and moments results in internal compressive, tensile, and shear stress [57]. However, the individual contribution of each stress component to the development of DTI is difficult to quantify.

So far, DTI research focussed mainly on compressive loading. Researchers have defined threshold levels for direct deformation damage, based on the relationship between pressure or compressive strain, and time [42, 43]. Subsequently, they compared these thresholds to computed values of internal compressive stress and strain in prosthetic patients to predict the likelihood of DTI development [30, 31, 33]. However, the results of these studies must be interpreted with caution.

Firstly, the threshold levels were deducted from experiments on animal tissue and bioartificial muscle constructs, and not intended to be translated directly to human studies [42, 43]. Secondly, the experimental design of threshold studies was oriented towards measuring direct deformation damage, whilst the microvascular and lymphatic system were mostly neglected. And lastly, the application of a static, compressive load differs from residuum biomechanics. Researchers showed that a cyclic force application may reduce the static threshold levels [40, 51, 58, 59], implying that the predicted risk of DTI in transtibial amputees has been underestimated. The addition of a shear component might also induce a greater damage potential by reducing the capillary closure pressure, which in turn increases the risk for ischemia [60, 61].

To minimise these limitations, new research approaches are needed. The effects of shear and distortion on tissues and cells should be examined with mechanical, microvascular, and lymphatic damage in mind. Additionally, researchers repeatedly stressed the need for a dynamic threshold level that combines compression, tension, and shear [29, 30, 33]. For this reason, our research group is working on investigating tissue response to a cyclic loading protocol under a variety of loading conditions that represent transtibial prosthetic use. This will allow for a better interpretation of existing data on internal loading conditions and contribute to the knowledge of DTI development.

#### Microvasculature and the lymphatic system

Our review revealed a gap in the aetiological research. Whilst the connection between deformation damage and prosthetic use has been established experimentally, the role of microvasculature and the lymphatic system was only discussed in general review papers.

Regarding the vascular system, the effects of a cyclic loading protocol with ischemic and reperfusion phases altering at high frequencies are unknown. The literature on similar loading scenarios is ambiguous: Some experimental results indicate an accumulation of cellular and tissue damage in comparison to continuous loading. However, the studies were either based on animal skin [58, 59, 62] or used prolonged time intervals [50, 51]; these were far below frequencies of about 1 Hz commonly seen during prosthetic gait [31, 63]. Other studies report on the adaptive capabilities of soft tissue [64–67], which indicates an increased tissue tolerance towards ischemic and reperfusion damage [68].

The structure and function of the lymphatic system and its response to loading are barely understood. In general, compression in combination with shear stress may affect lymph formation and propulsion [69]. The resulting dysfunction of lymphatic drainage can lead to an accumulation of toxic waste products, which damages cells and tissues [70, 71]. In the last decade, this theory was supported by a small number of studies. Researchers confirmed a reduction of lymphatic clearance during compressive loading [72], as well as toxic effects of accumulated bio-wastes on cell viability [73]. Reperfusion seems to add additional stress on the lymphatic system [74]. These factors might be aggravated in transtibial amputees. The initial amputation process causes traumatic injury to the lymphatic system [39, 75], followed by a reduced muscle tone post-amputation, which might reduce the propulsive capabilities of lymphatic vessels and results in oedema [76].

For a better understanding of the role of the vascular and lymphatic system in DTI development, the simulation of high-frequency cyclic loading protocols with perfused tissue will be necessary. Beside histological analysis of muscle damage in animal models, arterial spin labelling or dynamic contrast MRI could relate the damage to muscle perfusion and oxygenation [77]. For clinically oriented studies, near-infrared (NIR) spectroscopy is potentially capable of evaluating the oxidative function of transtibial residua [78, 79]. The lymphatic system could be explored in a similar fashion by visualising lymph flow with NIR fluorescent imaging [72]. Together with information from direct deformation studies, the results of microvascular and lymphatic experiments can feed into the prevention, diagnosis, and treatment of DTI.

### Population-specific risk factors

Identifying population-specific characteristics that may have a negative influence on the soft tissue in amputees is equally as important as estimating internal loading states. We found a variety of risk factors that link transtibial prosthetic use to DTI development, as detailed in the following paragraphs.

#### Influence of tissue characteristics

Our review identified a range of tissue-related determinants of DTI risk. Firstly, tissue morphology and geometry change as a result of the surgical process. By revisiting established amputation procedures and considering alternatives, for example the use of bone bridges [80] instead of bevelled bone edges, or different flap techniques that change the location of surgical scars [81, 82], some of these risk factors might be avoidable.

On the other hand, naturally occurring risk factors are hard to eliminate. The residuum commonly undergoes changes of intramuscular fat infiltration and muscular atrophy post-amputation [65, 83], which were both shown to enhance internal tissue deformation in SCI patients [13]. Additionally, amputees often experience complications after surgery like osteophytes or angular deformities [24, 25], which also have adverse effects on tissue loading [36, 39]. Overall, a multitude of amputation-related tissue characteristics aggravates the challenging loading conditions in transtibial amputees.

#### The role of co-morbidities

The main cause of transtibial amputation in the high-income countries is disvascularity. In the UK and the US, most patients suffer from diabetes and other peripheral vascular diseases [84–86]. This affects the perfusion of their residua, which facilitates ischemic damage and exacerbates inflammation. In diabetic patients, neuropathy is another common complication. The review papers we identified stressed that as the body’s protective pain mechanism is suspended, affected individuals may not distribute potentially damaging loads anymore [26, 40].

To validate this link between co-morbidities and DTI development in transtibial amputees, epidemiological studies would be beneficial. These studies also offer a chance to uncover possible correlations of other factors, such as time since amputation or age. Additionally, they could give an insight into the extent of the prevalence of DTI in transtibial amputees in general. The epidemiology of DTI in lower-income countries should also be investigated, as their amputation population profiles, surgical procedures, and prosthetic componentry differ significantly from high-income countries [86–88].

#### Prosthesis-related risk factors

Prosthetic sockets are in direct contact with the residuum. Hence, they are the component most frequently associated with DTI development. Researchers highlighted the influence of the socket shape on internal pressure distribution and soft tissue deformation [9, 40, 41]. However, studies that quantify differences in internal loading conditions between the two main socket designs PTB and TSB are missing. To close the gap, biomechanical and physiological studies could be conducted. This information would help to define good prosthetic fit and improve clinical practice.

Other prosthetic componentry like prosthetic feet and liners have also shown to influence the internal loading conditions [32, 38]. Before this information can be utilised reliably in clinical practice, more studies with large sample sizes and low variability are needed. This applies not only to studies on prosthesis-related risk factors, but to all experimental DTI studies on human subjects. Thus, consistency in patient groups, socket types, alignment, and componentry are crucial.

### Methodological approaches

Tissue loading is undoubtedly a major factor in the development of DTI. Traditionally, the interface pressure between the socket and the residuum was quantified with sensor technologies [40, 89]. With the discovery of deep tissue layers as the potential origin of pressure ulcers [4, 11], the focus shifted towards assessing internal mechanics. New methodologies were needed as the forces applied at the interface do not reflect the internal loading states [90, 91].

#### Computational and analytical modelling

According to our review, modelling was the measure of choice. Medical images, interface pressure measurements, and data on mechanical properties of soft tissue informed numerical and analytical models. Researches then used the estimated stress and strain values of those models to determine general loading patterns during various scenarios and patient-specific DTI risks.

However, the interpretation of estimated stress and strain values beyond basic trends is limited. This comes down to the complexity of soft tissue mechanics. Skeletal muscle shows time-dependent behaviour under load, including hysteresis, stress relaxation, and creep [92, 93]. Due to its cellular arrangement in parallel fibrous structures, the direction of load application alters the mechanical properties, known as anisotropy [92]. Additionally, the residuum consists not only of skeletal muscle, but also adipose tissue, connective tissue, and skin, which all have different, patient-specific mechanical properties. Each layer might also be affected by inhomogeneities, like scar tissue or fat infiltration. Taken together, the intricate nature of soft tissues exceeds the modelling capabilities of existing computational approaches [41]. The research on biomechanics of the residual limb is no exception and usually draws on simplified concepts with assumptions of linear elasticity, isotropy, or homogeneity. Even though clinical translatability is a core issue in DTI research, the accuracy of the proposed patient-specific modelling approaches based on these simplifications is questionable.

Nevertheless, promising methodological developments may increase the accuracy of future research efforts (Table 8). An additional advancement is the integration of model post-processing. Muscle stiffening is a phenomenon observed after the onset of DTI, which redistributes subsequent loads towards adjacent sites and promotes damage progression in a positive feedback loop [44, 99]. Integrating this damage law into FE models for prolonged loading increases the accuracy of DTI prediction [33, 44]. Widening this approach to model pre-processing by incorporating the effects of the donning procedure, as Lacroix and Patino [100] did for transfemoral amputees, would improve modelling even further.

**Table 8:** Potential technologies for advanced FE modelling input

Soft Tissue Property		Technology	Reference
Anisotropy		Diffusion Tensor MRI (DT MRI)	Ramsay et al. 2018 [94]
Inhomogeneity	Different soft tissue layers	MRI, Ultrasound	Bader and Worsley 2018 [95]
	Scar tissue	Elastography	Strijkers et al. 2019 [96]
	Fatty infiltration	Dixon MRI method, Magnetic Resonance Spectroscopy, T2 relaxation time mapping	Strijkers et al. 2019 [96]
Elasticity and stiffness		Elastography	Nelissen et al. 2017 [97] Sigrist et al. 2017 [98]

#### Alternatives to determine internal loading states

Modelling is not the only approach to determine internal loading states. Medical imaging offers ways to visualise soft tissue deformation directly. MRI tagging has been proposed and used to derive strain values of compressed muscle [96, 101]. Another option would be to measure physiological features that are indirectly related to the mechanical state of tissues. DT MRI for example captures changes in diffusivity due to compression [102], making it a potential measure for strain [103]. Given the complexity and limitations that the predictive nature of analytical and computational modelling inheres, it seems sensible to integrate technologies that have not yet been used in the context of pressure ulcer research.

### Limitations

Whilst we believe that our systematic approach according to the PRISMA-ScR guidelines identified most of the relevant literature, there are various sources of potential bias. Firstly, the lack of registration of the review protocol prior to performing this scoping review may have introduced conduction and reporting bias. By restricting the search to English language and excluding grey literature, we might have missed studies and introduced selection bias. Further papers might have been overlooked due to the heterogeneities in wording and definitions of pressure injuries [8, 104, 105], despite the use of common DTI- and prosthesis-related expressions in our search strategy. Additionally, the review process was mainly conducted by one reviewer. We aimed to limit possible selection bias by consulting a second reviewer in case of uncertainty. Lastly, we focussed on transtibial prosthetic users, whereas transfemoral amputees might experience similar conditions and could therefore be included in future endeavours.

## Conclusion

This scoping review recorded an overwhelming interplay of aetiological and risk factors for DTI in transtibial prosthetic users. It also highlighted the urgent need for fundamental research in this area. Particularly relevant is the unique biomechanical environment created by the interaction of the residuum and prosthetic socket during gait, which is characterised by high shear forces and repetitive loading. Combining the tissue’s response to dynamic loading, which is currently investigated by our research team, with advanced measurement methods like imaging techniques could provide new insights into the aetiology of DTI. A better understanding of the influence of prostheses on the vascular and lymphatic system is also essential.

To realise these endeavours effectively, an interdisciplinary approach and the integration of stakeholders is inevitable. The results of prosthesis-oriented research have the potential to provide the scientific basis for consensus on much-needed advancements in clinical practice: from improved amputation and rehabilitation processes, to updated socket design and fitting procedures, and the optimised use of componentry. A growing body of research on DTI in transtibial prosthetic users could also inform pressure ulcer categorisations, reporting standards, and international guidelines. Ultimately, the goal should be to reduce the prevalence and risk of DTI in transtibial prosthetic users and improve patient care.

## Methods

This scope review follows a methodological framework [27, 106], which was refined by the Joanna Briggs Institute (JBI) [107]. It is also compliant with the PRISMA-ScR checklist for scoping reviews [108] (see Additional file 1).

### Search strategy

The database search included Pubmed (Medline), Ovid Excerpta Medica (Embase classic and Embase), and Scopus. It covered the first possible date for each database until June 2019, when we conducted the search. We utilised automatized categorisation of the MeSH term “pressure ulcer” or a database-specific equivalent. This inclusion of the generic pressure ulcer term ensured full coverage, as DTI was only recently accredited as separate pressure injury category [5]. The full search strategy was constructed as follows (Table 9):

**Table 9:** Search Strategy explained on the example of Ovid Excerpta Medica

Search #	Search Term	Key Concepts
1	Deep Tissue Injury(*)	Deep Tissue Injury (DTI)
2	Deep Tissue Damage	
3	Decubitus/	
4	#1 OR #2 OR #3	
5	Transtibial	Amputation level
6	Trans-tibial	
7	Below knee	
8	#5 OR #6 OR #7	
9	Prosthe*	Transtibial prosthetic users
10	#8 AND #9	
11	#4 AND #10	DTI in transtibial prosthetic users

^*^represents the "wildcard" that is commonly used in literature search strategies

We scanned reference lists and forward-citation reports of eligible manuscripts for relevant articles that have not been identified in the automatic search. Additionally, we contacted an expert in the field of DTI for further literature suggestions.

### Study selection and inclusion criteria

Primary and secondary sources were retrieved from the automatic search. We filtered out duplicate articles and duplicates published as conference proceedings with the aid of a citation software (Zotero 5.0.69).

For further assessment, one reviewer (M.G.) screened the remaining articles. They had to be written in English, have an available full text, and have a title and abstract relevant to DTI as a result of prosthetic use. Studies on unrelated conditions and with amputations other than at transtibial level were excluded. Likewise, papers describing superficial, Stage 1, or Stage 2 pressure injuries were disregarded. To ensure a minimum qualitative standard, all papers had to be peer reviewed.

In the following full-text examination, one reviewer (M.G.) evaluated the relevance, study group characteristics, and type of pressure injury. Additionally, transtibial prosthetics and DTI had to be the focus of the article. In case of uncertainty about eligibility of articles, a second reviewer (A.B.) was consulted and possible disagreements discussed until consensus was reached.

By scanning through reference lists and forward-citations of eligible papers, we identified further resources. We validated their suitability with the previously described process, before including them in the final qualitative synthesis. We also retrieved recommendations on relevant literature from expert contact. All but one of the suggested sources were already included in the current synthesis, with the additional paper being irrelevant for this review.

We extracted data from the combined search results according to suggestions by the JBI [107] and the PRISMA-ScR checklist [108] with slight adjustments. The variables of interest were author, year, country, type of study, aims and objectives, study population and sample size (if applicable), methodology, outcome measures, and key findings related to DTI in transtibial prosthetic users. The complete range of studies was then thematically grouped for further in-depth analysis, which is presented in the “Results” section in tables and supporting narratives.

## Supplementary information

**Additional file 1.** PRISMA-ScR Checklist. Preferred Reporting Items for Systematic reviews and Meta-Analyses extension for Scoping Reviews (PRISMA-ScR) Checklist.

## Data Availability

All data generated or analysed during this study are included in this published article and its supplementary information files.

## Abbreviations

DT MRI: Diffusion Tensor MRI
DTI: Deep Tissue Injury
ESR: Energy storage and return
FE: Finite element
JBI: Joanna Briggs Institute
MDRPU: Medical device related pressure ulcer
MRI: Magnetic resonance imaging
NIR: Near infrared
NPUAP: National Pressure Ulcer Advisory Panel
PTB: Patellar tendon bearing
RMS: Root mean square
SCI: Spinal cord injury
SED: Strain energy density
TSB: Total surface bearing
TTA: Transtibial amputee
